# Lymphocyte-to-monocyte ratio associated with severe post-stenotic aortic dilation in a case–control study

**DOI:** 10.1186/s12872-022-02636-3

**Published:** 2022-04-26

**Authors:** Shanghao Chen, Zezhong Wu, Yan Yun, Hechen Shen, Diming Zhao, Yanwu Liu, Chengwei Zou, Haizhou Zhang, Zhengjun Wang, Xiaochun Ma

**Affiliations:** 1 Department of Cardiovascular Surgery, Shandong Provincial Hospital, Cheeloo College of Medicine, Shandong University, No.324 Jingwu Road, Jinan, 250021 Shandong China; 2 Department of Cardiovascular Surgery, Shandong Provincial Hospital Affiliated to Shandong First Medical University, No.324 Jingwu Road, Jinan, 250021 Shandong China; 3 Department of Radiology, Qilu Hospital of Shandong University, No. 107 West Wenhua Road, Jinan, 250012 Shandong Province China

**Keywords:** Lymphocyte-to-monocyte ratio, Post-stenotic aortic dilation, Association

## Abstract

**Background:**

Calcific aortic valve stenosis (CAVS) represents a serious health threat to elderly patients. Post-stenotic aortic dilation, a common feature in CAVS patients, might progress into aneurysm and even dissection, potential consequences of CAVS, and predicts a poor prognosis. This study sought to investigate the association of lymphocyte-to-monocyte ratio (LMR), an inflammatory biomarker, with severe post-stenotic aortic dilation in a case–control study in Chinese population.

**Materials and methods:**

208 consecutive patients with CAVS were recruited retrospectively in a case–control study in Chinese population, from July 1, 2015 to June 31, 2018. LMR was statistically analyzed using the ROC curve and binary logistic regression analyses for its prognostic value in severe post-stenotic aortic dilation.

**Results:**

LMR was significantly reduced in patients with severe post-stenotic aortic dilation (2.72 vs. 3.53, *p* = 0.002 < 0.05) compared to patients without severe post-stenotic aortic dilation. There was an inverse correlation observed between the maximal diameter of ascending aorta and LMR in the overall patients (r = − 0.217, *p* = 0.002 < 0.05). For post-stenotic aortic dilation, the prevalence of high-LMR group was statistically lower than that of low-LMR group (19.7% vs. 43.9%, *p* < 0.001). The maximal diameter of ascending aorta was significantly reduced in the high-LMR group (4.35 vs. 4.76, *p* = 0.003 < 0.05) compared to low-LMR group. Additionally, LMR was identified in the multivariate analysis independently associated with severe post-stenotic aortic dilation (AUC 0.743, 95% CI: [0.573–0.964], *p* = 0.025).

**Conclusions:**

This study provided the evidence of an inverse correlation between severe post-stenotic aortic dilation and LMR. LMR is potentially independently associated with severe post-stenotic aortic dilation.

**Supplementary Information:**

The online version contains supplementary material available at 10.1186/s12872-022-02636-3.

## Introduction

Calcific aortic valve stenosis (CAVS) is the most prevailing heart valve disorder and affects nearly 1.7% in the population > 65 years old in developed countries [1]. As the population ages, the prevalence has also rapidly increased in developing countries [2]. Post-stenotic aortic dilation is common in patients with CAVS and might advance into aortic aneurysm and dissection [3, 4]. Thus, post-stenotic aortic dilation warrants regular medical monitoring to prevent dissection or rupture. In the management of patients with CAVS, it is necessary to decipher additional risk for post-stenotic aortic dilation, in an attempt to optimize the assessment of dilation progression, and clinical decision-making of a combined aorta replacement surgery. However, current data are scarce regarding the determinants and predictors of post-stenotic aortic dilation.

The pathophysiology of post-stenotic aortic dilation remains largely undetermined. Current evidence supported that this entity of aortic dilation might be due to the unfavorable haemodynamic conditions downstream of stenotic valves, or due to the valve anatomy (BAV and tricuspid aortic valve [TAV]) as well as the intrinsic pathology of aorta [5]. Given a pivotal role of inflammation widely recognized in the pathogenesis of CAVS, inflammation might also be linked with the development of post-stenotic aortic dilation. Of note, several inflammatory diseases as well as inflammatory biomarkers have been correlated with aortic dilatation, suggesting the effects of systemic and localized inflammation on the pathophysiology [6–9].

Lymphocyte-to-monocyte ratio (LMR) is an inflammatory biomarker calculated from peripheral blood count of lymphocytes and monocyte/macrophages. It has recently been associated with severity of coronary artery disease and prognosis following coronary artery bypass graft and percutaneous coronary intervention [10–12]. Besides, LMR has also been linked with the severity of rheumatic mitral valve stenosis, death prediction in heart failure and survival after endovascular repair of abdominal aortic aneurysms [13–15]. Here we hypothesized that LMR might function as a predictive indicator of severe post-stenotic aortic dilation and aimed to confirm this hypothesis in a case–control study in Chinese population.

## Material and methods

### Participants and study design

This study was a single-center retrospective case–control study. It was approved by the Ethics Committee of Shandong Provincial Hospital affiliated to Shandong First Medical University and Shandong University and the approval code for this study is NSFC2018-002. Due to its retrospective design, the written informed consents had to be waived. The study was performed in accordance with the Good Clinical Practice (GCP) and principles of the Declaration of Helsinki [16]. 208 patients were consecutively included in the study from the hospital above-mentioned, from July 1, 2015 to June 31, 2018. The time interval of this study was based on its funding which spanned roughly a similar period. All the included patients met the following criteria: (1) older than 18 years; (2) having a clear diagnosis of CAVS based on the criteria by the American College of Cardiology (ACC) and the American Heart Association (AHA). The subjects were excluded due to the following exclusion criteria: (1) the presence of rheumatic aortic valve stenosis or insufficiency, severe stenosis or insufficiency of mitral or tricuspid valves, endocarditis, coronary artery disease, atrial fibrillation, autoimmune diseases, malignancies and renal, hepatic or hematologic disorders; (2) suspicious findings of genetic tests or physical exam and family history suggesting a connective tissue disease; (3) whose related data were not sufficient. All conditions have been excluded that possibly affect these cell lines, especially the lymphocytes and monocytes. HIV infection has been excluded because all patients have been tested negative and corticosteroid administration has been similar between the two groups (Table 1). Although lymphopenia due to viral infections was hard to be excluded, no record of possible symptoms or administration of anti-viral drugs has been found. The recruited patients underwent a comprehensive evaluation of transthoracic echocardiography (TTE) and aortic CT angiography (CTA). The blood counts were measured and calculated for LMR before the aortic CTA or TTE was performed. Full blood counts were routinely collected for calculating LMR by dividing the number of lymphocytes by the number of monocytes.

**Table 1:** Clinical Characteristics of Patients with post-stenotic aortic dilatation and control group

	Post-stenotic aortic dilatation	Control group	*p* value
Patient population (n)	57	151	
Demographic data			
Age (years)	60.0 (11.0)	60.0 (12.0)	0.909
Sex, male (n)	41 (71.9%)	94 (62.3%)	0.174
Medical history			
Hypertension (n)	19 (33.3%)	56 (37.1%)	0.638
Smoking (n)	31 (54.4%)	63 (41.7%)	0.094
Diabetes (n)	3 (5.3%)	15 (9.9%)	0.435
Tricuspid aortic valve (n)	34 (59.6%)	79 (52.3%)	0.321
Chronic use of glucocorticoid (n)	21 (36.8%)	62 (41.1%)	0.636
Baseline echocardiography			
LVEDD (cm)	6.02 (1.74)	5.45 (1.49)	0.011
LVEF (%)	57 (7.5)	60 (7)	0.006
Laboratory tests			
Leukocyte (10^9^/L)	6.11 (2.06)	5.89 (1.96)	0.405
Neutrophil (10^9^/L)	3.48 (1.54)	3.49 (1.51)	0.896
Platelet (10^9^/L)	178 (75.5)	195.5 (71.5)	0.183
Monocyte (10^9^/L)	0.59 (0.37)	0.50 (0.24)	0.006
Lymphocyte (10^9^/L)	1.74 (0.68)	1.84 (0.68)	0.675
LMR	2.72 (1.62)	3.53 (1.75)	0.002
LDL-C (mmol/L)	2.78 (1.15)	2.80 (1.19)	0.843
CRP (mg/L)	0.93 (0.91)	0.91 (1.62)	0.388
MPV (fl)	10.60 (1.15)	10.90 (1.25)	0.329

The categorical variables in the table are presented by the number of cases (with percentage) and the continuous variables are expressed by the median (with interquartile range) or mean (with standard deviation)

*p* values were the results of unpaired t-test or Mann–Whitney U test for continuous variables, and χ^2^ test or Fisher’s exact test for categorical variables

*p* value: Compare the patients with and without ascending aorta dilatation

*LVEDD* left ventricular end diastolic diameter, *LVEF* left ventricular ejection fraction, *LMR* lymphocyte-to-monocyte ratio, *LDL-C* low density lipoprotein cholesterol, *CRP* C-reaction protein, *MPV* mean platelet volume

### The definition of severe post-stenotic aortic dilation

The maximal diameter of ascending aorta was measured by aortic CTA and severe post-stenotic aortic dilation was defined as the maximal diameter equal to or greater than 50 mm [17].

### The calculation of LMR and grouping by LMR

LMR was measured according to the lymphocyte and monocyte counts in the blood routine test. The cut-off LMR was decided by the diagnostic test as described below. The included patients were accordingly grouped as the low-LMR group (with their LMR values less than the cut-off values) and high-LMR group (with their LMR values greater than the cut-off values).

### Statistical analysis

All statistical analysis was performed using the software SPSS Statistics 25.0. The continuous variables were present in the form of mean ± standard deviation (SD) if the data conforms to normal distribution, or expressed as median (quartile deviation) if the data inconsistent with the normal distribution. The categorical variables were shown as frequencies (n) with percentages (%). For analyzing the continuous variables, the Student t-test was used when the normal distribution was conformed. Otherwise, the non-parametric Mann–Whitney U test was applied if a skewed distribution was met. The Chi-square or Fisher’s exact test was carried out for analyzing the categorical variables. The cut-off LMR was determined by the diagnostic tests using the receiver operating curve (ROC). The binary logistic regression analysis was employed for the univariable and multivariable analyses. When the efforts were made to construct a multivariable predictive model, all candidate variables derived from the univariable analysis (with a *p* value less than 0.1) as well as those possible predictive variables were selected. In logistic regression model, variables with *p* < 0.10 in the univariate model were entered into a multivariable predictive model and Forward: LR was undertaken with the probability of entry and removal as 0.10 and 0.20, respectively. A two-sided *p* value less than 0.05 was considered statistically significant.

## Results

### Characteristics of patients

#### Inclusion of patients

**Fig. 1:**
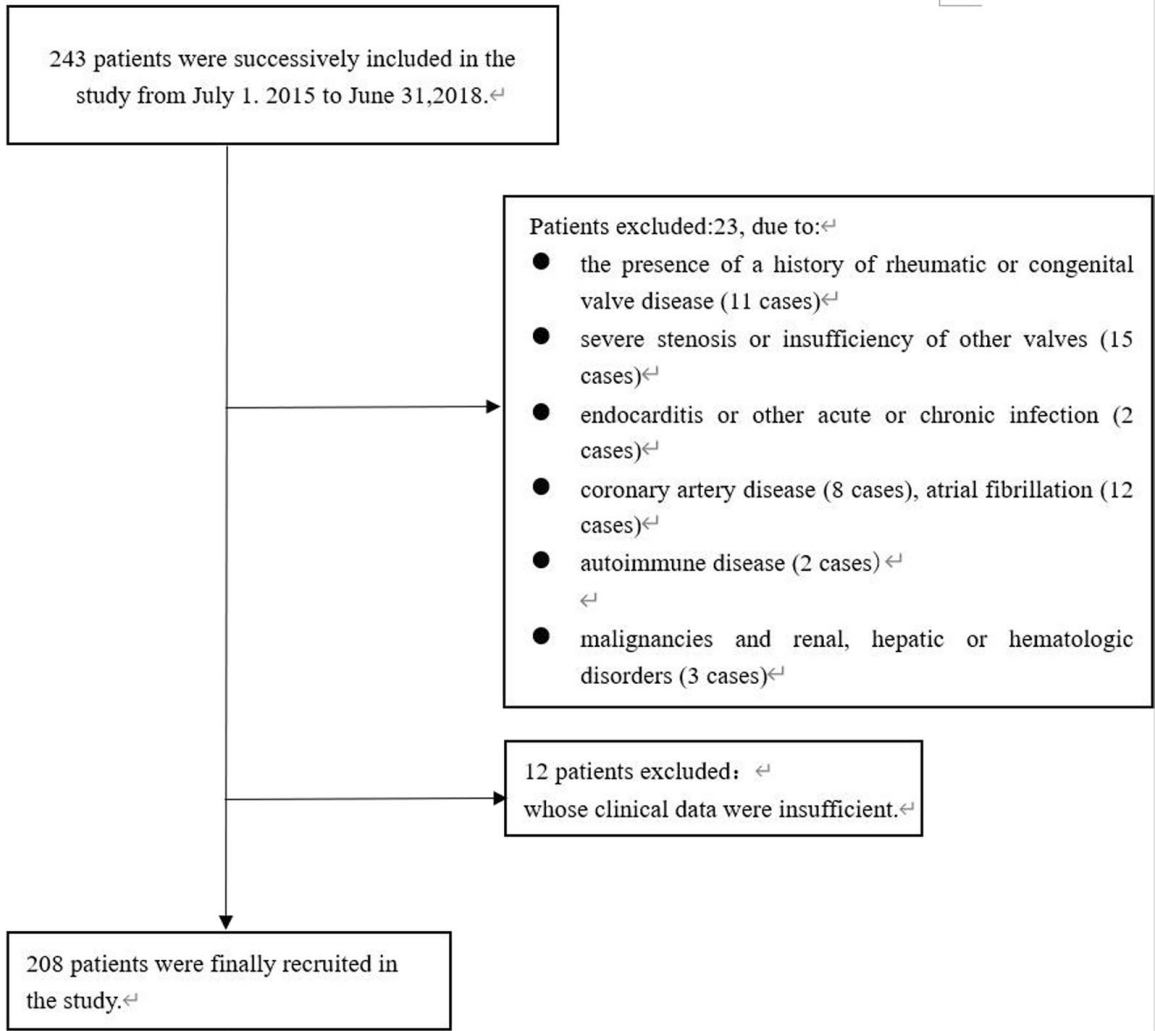
Flow diagram of exclusion and enrollment of study patients. Figure describes the exclusion and enrollment of study patients

From July 1, 2015 to June 31, 2018, 243 patients were recruited in sequence. 23 subjects were excluded because of the presence of rheumatic aortic valve stenosis (11 cases), severe stenosis or insufficiency of other valves (15 cases), endocarditis (2 cases), coronary artery disease (8 cases), atrial fibrillation (12 cases), autoimmune disease (2 cases), malignancies and renal, hepatic or hematologic disorders (3 cases); 12 patients excluded whose clinical data were insufficient (Fig. 1).

#### Detailed information of patients

For the patients with severe post-stenotic aortic dilation, 71.9% were male patients with a median age of 60.0 (11.0) years. 54.4% of patients were cigarette smokers, 33.3% had hypertension and 5.3% were with type 2 diabetes. 59.6% of patients had tricuspid aortic valve. The median LVEF was 57.0 (7.5). For the patients without severe post-stenotic aortic dilation, 62.3% were male patients with a median age of 60.0 (12.0) years. 41.7% of patients were cigarette smokers, 37.1% had hypertension and 9.9% were with type 2 diabetes. 52.3% of patients had tricuspid aortic valve. The median LVEF was 60.0 (7.0). The detailed information of patients with and without post-stenotic aortic dilation were summarized in the Table 1.

### LMR and severe post-stenotic aortic dilation

#### LMR in patients with severe post-stenotic aortic dilation or not

LMR was significantly reduced in patients with severe post-stenotic aortic dilation (2.72 vs. 3.53, *p* = 0.002 < 0.05) compared to patients without severe post-stenotic aortic dilation.

#### The correlation of LMR and maximal diameter of ascending aorta

**Fig. 2:**
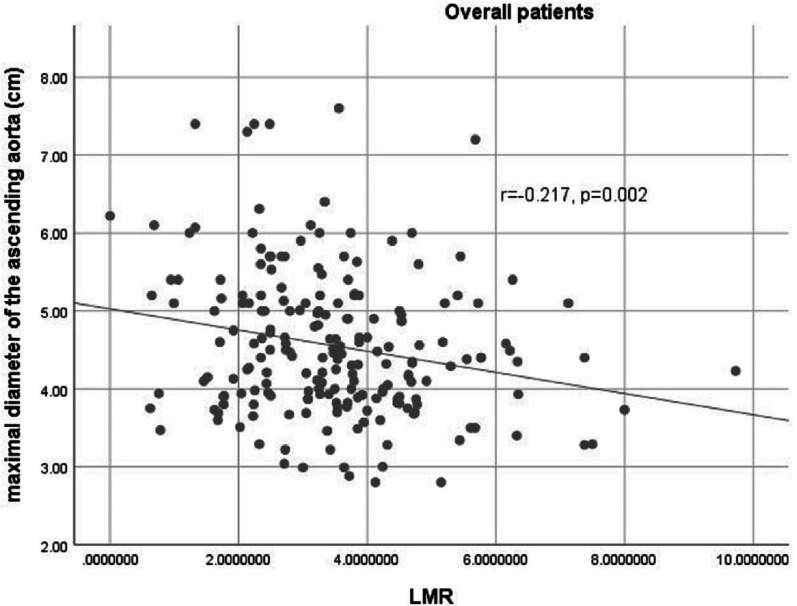
The correlation of LMR and maximal diameter of ascending aorta. Figure shows the inverse correlation between the maximal diameter of ascending aorta and LMR in the overall patients (r = − 0.217, *p* = 0.002 < 0.05). *LMR* lymphocyte-to-monocyte ratio

There was an inverse correlation observed between the maximal diameter of ascending aorta and LMR in the overall patients (r = − 0.217, *p* = 0.002 < 0.05) (Fig. 2).

#### Severe post-stenotic aortic dilation in the high-LMR and low-LMR groups

**Fig. 3:**
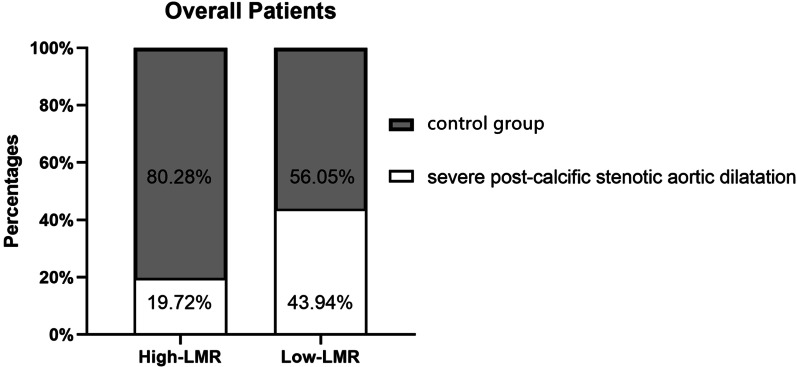
The prevalence of severe post-stenotic aortic dilation in the high-LMR and low-LMR groups. Figure depicts the prevalence of severe post-stenotic aortic dilation in the high-LMR and low-LMR groups. *LMR* lymphocyte-to-monocyte ratio

The cut-off LMR was determined by the diagnostic tests using the receiver operating curve (ROC) (AUC 0.640, 95% CI: [0.552–0.728], *p* = 0.002). When the value of sensitivity + specificity—1 reaches the maximum, the cut-off LMR was determined, which is 2.72 (Additional file 1: Fig. S1). Accordingly, the subjects were assigned into the high-LMR (n = 142) and low-LMR group (n = 66). The detailed information of two groups was summarized in Table 2. For severe post-stenotic aortic dilation, the prevalence of high-LMR group was statistically lower than that of low-LMR group (19.7% vs. 43.9%, *p* < 0.001). The maximal diameter of ascending aorta was significantly reduced in the high-LMR group (4.35vs. 4.76, *p* = 0.003 < 0.05) compared to low-LMR group (Fig. 3).

**Table 2:** Clinical characteristics of patients with high-LMR or low-LMR

	High-LMR	Low-LMR	*p* value
Patient population (n)	142	66	
Demographic data			
Age (years)	59 (13)	63 (8.25)	0.005
Sex, male (n)	86 (60.6%)	49 (74.2%)	0.048
Medical history			
Hypertension (n)	42 (29.6%)	33 (50.0%)	0.004
Smoking (n)	62 (43.7%)	32 (48.5%)	0.488
Diabetes (n)	14 (9.9%)	4 (6.1%)	0.372
Post-calcific stenotic aortic dilatation (n)	28 (19.7%)	29 (43.9%)	< 0.001
Maximal diameter of the ascending aorta (cm)	4.35 (1.03)	4.76 (1.60)	0.003
Tricuspid aortic valve(n)	61 (43.0%)	52 (78.8%)	< 0.001
Baseline echocardiography			
LVEDD (cm)	5.41 (1.43)	6.29 (1.41)	< 0.001
LVEF (%)	60 (7)	58 (7.5)	0.031
Preoperative laboratory tests			
Leukocyte (10^9^/L)	5.74 (1.93)	6.16 (1.90)	0.020
Neutrophil (10^9^/L)	3.33 (1.48)	3.73 (1.26)	0.004
Platelet (10^9^/L)	193 (69)	187 (85.25)	0.818
Monocyte (10^9^/L)	0.47 (0.20)	0.79 (0.43)	< 0.001
Lymphocyte (10^9^/L)	1.88 (0.51)	1.54 (0.67)	< 0.001
LDL-C (mmol/L)	2.87 (1.18)	2.68 (1.12)	0.101
CRP (mg/L)	0.77 (1.43)	1.18 (2.07)	0.134
MPV (fl)	10.90 (1.30)	10.60 (1.15)	0.287

The categorical variables in the table are presented by the number of cases (with percentage) and the continuous variables are expressed by the median (with interquartile range) or mean (with standard deviation)

*p* values were the results of unpaired t-test or Mann–Whitney U test for continuous variables, and χ2 test or Fisher’s exact test for categorical variables

*p* value: Compare the overall patients with Low-LMR or High-LMR

*LVEDD* left ventricular end diastolic diameter, *LVEF* left ventricular ejection fraction, *LMR* lymphocyte-to-monocyte ratio, *LDL-C* low density lipoprotein cholesterol, *CRP* C-reaction protein, *MPV* mean platelet volume

### LMR associated with severe post-stenotic aortic dilation

Multivariable analysis of risk factors for severe post-stenotic aortic dilation was carried out by including all possible predictive variables (including age, sex, hypertension, diabetes, smoking, LMR, left ventricular ejection fraction, neutrophil, platelet, C-reaction protein, low-density lipoprotein cholesterol and tricuspid aortic valve) [18, 19]. The results showed that LMR was associated with severe post-stenotic aortic dilation in Table 3 with a satisfied ROC curve (AUC 0.743, 95% CI: [0.573–0.964], *p* = 0.025) (Additional file 1: Fig. S2).

**Table 3:** Multivariate analysis of risk factors for severe post-stenotic aortic dilation

Variables	B	SE	*p* value	OR	95% CI
Age	0.057	0.017	0.041	1.059	1.023–1.095
LMR	− 0.371	0.128	0.004	0.690	0.537–0.886
Hypertension	0.284	0.340	0.034	1.329	1.257–1.401
Constant	2.218	1.183	0.018	1.243	

Multivariate analysis of risk factors for severe post-stenotic aortic dilation were performed. *p* values were the results of binary logistic regression analysis

*LMR* lymphocyte to monocyte ratio, *OR* odds ratio, *CI* confidence interval, *SE* standard error

## Discussion

In this case–control study, 208 patients were enrolled and the correlation between LMR andsevere post-stenotic aortic dilation was analyzed. The results presented the evidence of a lower LMR in the patients with severe post-stenotic aortic dilation compared to the patients without post-stenotic aortic dilation. The prevalence of severe post-stenotic aortic dilation in the high-LMR group was statistically lower than that in the low-LMR group. Finally, LMR was considered associated with severe post-stenotic aortic dilation after adjusting other possible variables.

Severe post-stenotic aortic dilation is often clinically silent until its complications, acute aortic dissection or rupture, occurs [20]. Although Davies and colleagues demonstrated that the ascending aorta with a diameter within 35–39 mm is not associated with aortic rupture or dissection, the risk of rupture or dissection increased dramatically by 27-fold if the diameter of ascending aorta reaches 60 mm or more [21]. Thus, it is a clinical priority to identify the predictive biomarkers or modifiable risk factors in the prevention and treatment of severe post-stenotic aortic dilation.

The associated risk factors for post-stenotic aortic dilation in the previous reports were mainly male gender, bicuspid aortic valve, hypertension and smoking [3, 22, 23]. Aortic dilatation has been linked with inflammatory diseases such as infectious aortitis, Takayasu arteritis, and giant cell arteritis [7, 8, 24]. While levels of several inflammatory biomarkers such as C-reactive protein (CRP) and interleukin (IL)-6 are elevated in patients with thoracic aortic aneurysms, suggesting the effects of systemic inflammation on the pathophysiology [6]. Of note, an increased activity of matrix metalloproteinases (MMPs) has been described in the media of thoracic aortic aneurysm, a characteristic of localized inflammation [9, 25]. Recent evidence from basic research has also suggested the role of several canonical inflammatory signaling pathways, AP-1 and the ERK1/2 signaling pathway, in contributing to the inflammation in post-stenotic aortic dilation [26]. However, the detailed mechanisms underlying the inflammatory reaction in post-stenotic aortic dilation is still unclear. And it is still currently unclear which inflammatory cell set is primarily responsible for the inflammatory reaction in post-stenotic aortic dilation. Thus, identifying feasible inflammatory biomarkers will be constructive in predicting the high-risk patients and supportive in early diagnosis and intervention.

LMR is a measure calculated from the exact number of lymphocytes and monocytes in the peripheral blood. As an inflammation-related indicator, a lower LMR appears to be associated with decreased survival and increased recurrence in malignancies [27]. LMR has recently been emphasized associated with severity of coronary artery disease and prognosis following coronary artery bypass graft and percutaneous coronary intervention [10–12]. Besides, LMR has also been linked with the severity of rheumatic mitral valve stenosis, death prediction in heart failure. and survival after endovascular repair of abdominal aortic aneurysms [13–15]. Our findings demonstrated that LMR is significantly lower in the patients with severe post-stenotic aortic dilation compared to the patients without post-stenotic aortic dilation. An inverse correlation was observed between the maximal diameter of ascending aorta and LMR. It indicated that as the maximal diameter of ascending aorta develops from the normal status to dilatation, LMR might reduce proportionally. Thus, the patients with CAVS having a lower LMR could be potentially categorized into the high-risk population of post-stenotic aortic dilation. And this parameter is potentially applied to identify the patients with mild to moderate aortic dilation, follow-up the disease progression as well as disease stratification. Due to the narrow treatment time window for aortic aneurysm and dissection, early diagnosis and prognostic assessment is essential. Early recognition of patients in higher risk of post-stenotic aortic dilation would allow medical staffs to take timely measures to prevent the occurrence of aortic dissection and even rupture. With the aid of risk stratification of post-stenotic aortic dilation by LMR, early identification of patients at high risk might be realized, which would lead to the close monitoring and early initiation of efficient preventive and therapeutic strategies. Besides, a fraction of patients with post-stenotic aortic dilation is asymptomatic until aortic dissection or rupture occurs. For this group of patients, aortic CTA is not often included in routine physical examination and follow-up. Therefore, LMR might be easier and cheaper for follow-up of these patients. A more accurate cut-off LMR should be confirmed by more prospective studies with larger sample sizes, in order to aid in risk-stratification in the future.

## Limitations

Several limitations were present and special attention should be paid to interpret the results in this study. First of all, the study design, a single-center retrospective observational study, inevitably introduced a source of potential bias. The second limitation lies in a small simple size. Third, some confounding variables, such as other inflammatory markers, might influence the role of LMR in post-stenotic aortic dilation. Fourth, the association of LMR and mid- and long-term endpoints was not investigated. Fifth, the case–control design of this study did not evaluate the causal relationships between LMR and post-stenotic aortic dilation. Sixth, the exclusion criteria restricted the selection of population and excluded the patients with other common cardiovascular conditionsto isolate an effect of LMR on outcomes. It might make the results only applicable in some clinical settings.

## Conclusions

LMR is inversely correlated with the prevalence and severity of post-stenotic aortic dilation and might be associated with post-stenotic aortic dilation. Further large-scale case–control studies are needed to confirm these results in the future.

## Supplementary Information

**Additional file 1. Fig. S1**. ROC curve of diagnostic tests in cut-off LMR. **Fig. S2**. ROC curve of logistic regression.

## Data Availability

The datasets used and analyzed during the current study are available from the corresponding author on reasonable request.

## Abbreviations

CAVS: Calcific aortic valve stenosis
BAV: Bicuspid aortic valve
TAV: Tricuspid aortic valve
LMR: Lymphocyte-to-monocyte ratio
GCP: Good clinical practice
ACC: American College of Cardiology
AHA: American Heart Association
TTE: Transthoracic echocardiography
CTA: CT angiography
SD: Standard deviation
ROC: Receiver operating curve
CRP: C-reactive protein
MMPs: Matrix metalloproteinases
