# Supplementary material for: Lymphocyte-to-monocyte ratio associated with severe post-stenotic aortic dilation in a case–control study
Source: BMC Cardiovasc Disord. 2022 Apr 26;22:195. doi: 10.1186/s12872-022-02636-3 (PMC9044758; doi:10.1186/s12872-022-02636-3)
Supplement: Supplementary file 1 — Additional file 1. Fig. S1. ROC curve of diagnostic tests in cut-off LMR. Fig. S2. ROC curve of logistic regression. [file 12872_2022_2636_MOESM1_ESM.docx]

**Supplementary figure 1 ROC curve of diagnostic tests in cut-off LMR.**

Supplementary figure 1 shows ROC curve (AUC 0.640, 95% CI: [0.552-0.728], p=0.002) of diagnostic tests in cut-off LMR.

ROC, Receiver Operating Curve; LMR, Lymphocyte-to-Monocyte Ratio.


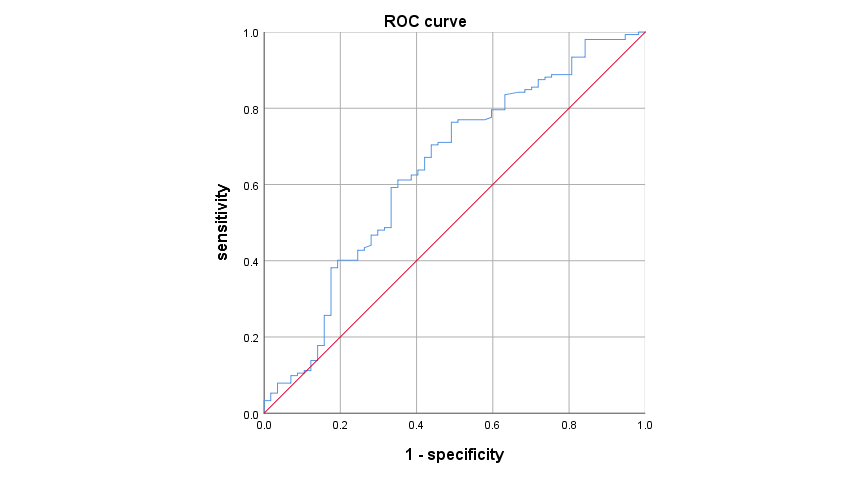


AUC 0.640, 95% CI: [0.552-0.728], p=0.002

**Supplementary figure 2 ROC curve of logistic regression.**

Supplementary figure 1 shows ROC curve (AUC 0.743, 95% CI: [0.573-0.964], p=0.025) of logistic regression.

ROC, Receiver Operating Curve.


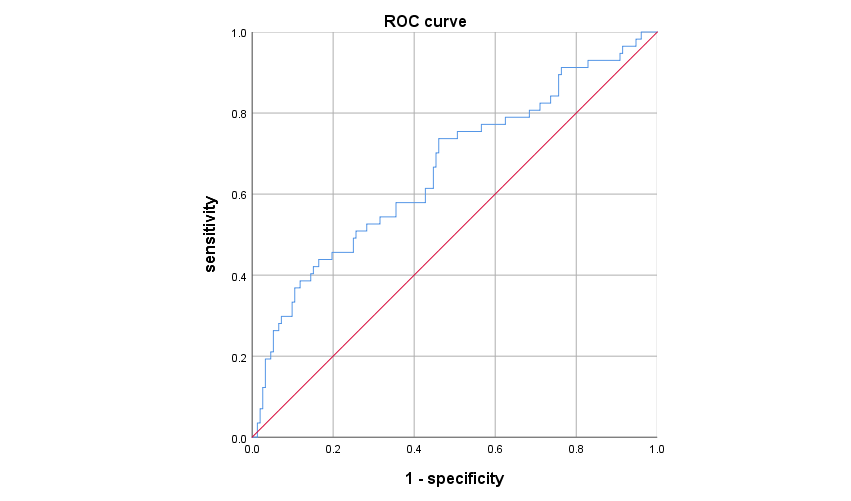


AUC 0.743, 95% CI: [0.573-0.964], p=0.025
